# Degeneration in ACL Injured Knees with and without Reconstruction in Relation to Muscle Size and Fat Content—Data from the Osteoarthritis Initiative

**DOI:** 10.1371/journal.pone.0166865

**Published:** 2016-12-05

**Authors:** Pia M. Jungmann, Thomas Baum, Michael C. Nevitt, Lorenzo Nardo, Alexandra S. Gersing, Nancy E. Lane, Charles E. McCulloch, Ernst J. Rummeny, Thomas M. Link

**Affiliations:** 1 Department of Radiology, Technische Universitaet Muenchen, Munich, Germany; 2 Department of Epidemiology and Biostatistics, University of California San Francisco, San Francisco, California, United States of America; 3 Musculoskeletal and Quantitative Imaging Research, Department of Radiology and Biomedical Imaging, University of California San Francisco, San Francisco, California, United States of America; 4 Department of Internal Medicine, UC Davis Medical Center, Sacramento, California, United States of America; Semmelweis Egyetem, HUNGARY

## Abstract

**Background:**

Anterior cruciate ligaments (ACL) injuries represent a major risk factor for early osteoarthritis (OA).

**Purpose:**

To evaluate the prevalence and 4-year progression of knee OA measured with 3T MR-imaging in individuals with ruptured, reconstructed or normal ACL and to assess the impact of thigh muscle characteristics.

**Methods:**

A total of 54 knees (23/54 male, 31/54 female) were recruited from the Osteoarthritis Initiative (OAI). At baseline, 15/54 subjects had prevalent ACL ruptures and 15/54 subjects had prevalent ACL reconstruction (24/54 normal ACL). Western Ontario and McMasters Universities Arthritis Index (WOMAC) scores, Physical Activity Scores of the Elderly (PASE) and thigh muscle characteristics including strength, fat infiltration (Goutallier score) and thigh muscle cross-sectional area (CSA) MR measurements were obtained at baseline. Whole-organ MR-imaging Scores (WORMS) were obtained at baseline and at a 4-year follow-up time-point. Multivariate regression models, adjusting for covariates (age, gender, body mass index), were used for statistical analysis.

**Results:**

At baseline, subjects with prevalent ACL ruptures had worse WORMS total scores (mean±SEM, 44.1±3.5) than subjects with ACL reconstruction (30.8±4.0; P = 0.015) and worse than subjects with normal ACL (21.3±3.0; P<0.001). Cartilage scores were worse in both femorotibial compartments in ACL injured knees than in knees with normal ACL (P<0.05). Knees with ACL reconstruction showed an increased degeneration of the medial meniscus (P = 0.036), cartilage degeneration at the medial femoral condyle (P = 0.011). In a multivariate regression model, including both ACL groups and total muscle characteristics as influence parameters, high thigh muscle CSA, high muscle/ fat ratio and low Goutallier scores were associated with less degenerative changes at the knee, independent of ACL status. Knees with ACL reconstruction showed an increased progression of cartilage degeneration at the medial tibia compared to the normal ACL group (P = 0.027).

**Conclusions:**

High thigh muscle CSA is associated with less degenerative changes at the knee, independent of the ACL status and may potentially be advantageous in the prevention of early OA.

## Introduction

Anterior cruciate ligament (ACL) ruptures are associated with an increased risk for post-traumatic osteoarthritis (OA) [1, 2]. Conservatively treated patients often cannot return to the level of their previous activities [3]. To prevent early OA, in approximately two-thirds of patients with ACL ruptures, treatment consists of ACL reconstruction, aiming to restore mechanical stability [2, 4, 5]. Most long-term studies report good results ten or more years after surgery [6, 7]. On the other hand, it has not been shown that surgical treatment reduces the risk for OA [8, 9].

Quadriceps weakness, high muscle volume and other muscle parameters are known to be associated with knee joint degeneration [10, 11]. Both, muscle characteristics of the thigh and knee joint degeneration may be determined quantitatively or semi-quantitatively using MR imaging. The muscle cross-sectional area (CSA) has been used to study muscle atrophy and hypertrophy on MR imaging. [12, 13]. Using CSA, the vastus lateralis (VL)/ vastus medialis (VM) ratio has been shown to correlate with knee joint degeneration [14]. In comparison to conventional radiography, MR imaging allows for specific evaluation of the different joint structures and for detection of early OA [15]. There are several different MR imaging scores that aim to semi-quantitatively describe early degenerative changes of the knee joint, of which whole-organ magnetic resonance imaging score (WORMS) is the most frequently applied score [16–18]. Although many studies evaluate outcome after ACL reconstruction, no MR imaging study evaluates the impact of ACL status and thigh muscle characteristics on the longitudinal progression of degenerative changes at the knee [19].

The Osteoarthritis Initiative (OAI) is an ongoing longitudinal, NIH initiated multi-center cohort study with nearly 5000 participants. The aim is to provide a public research resource for investigation of the natural evolution of knee and hip OA and to identify potential prevention strategies. The OAI dataset includes clinical data, radiographs, bilateral 3T knee MRI studies and bilateral thigh MRI studies [20].

The purpose of the present study was to evaluate presence and 4-year progression of OA, measured with 3T MR imaging, in individuals with ruptured, reconstructed or normal ACL and the impact of thigh muscle area and other muscle characteristics. We hypothesized that not only ACL reconstruction but also muscle characteristics have an impact on long-term knee OA prevalence and progression in individuals with ACL injuries.

## Materials and Methods

### Subjects

Specific OAI datasets used in this study were medhist00 dataset, baseline datasets 0.2.2 and 0.E.1 and 4 year follow-up datasets 6.2.1 and 6.E.1 (http://www.oai.ucsf.edu/). The study protocol, amendments, and informed consent documentation were approved by the local institutional review boards. All clinical investigations have been conducted according to the principles expressed in the Declaration of Helsinki. Individuals from the OAI cohort with ACL reconstruction at baseline were identified by searching for the parameter”ever have ligament repair surgery”and by additional screening of the corresponding MR images for ACL reconstruction.

A subset of individuals from the OAI progression cohort (n = 304) was randomly selected. The progression cohort is characterized by the presence of symptomatic OA in at least one knee. Symptomatic OA is defined as the presence of pain, aching or stiffness in or around the knee on most days for at least 1 month during the past 12 months plus femorotibial osteophytes in the same knee on conventional radiographs. These knee MRIs were screened for the presence of complete ACL tear and for the presence of normal ACL. Individuals with mucoid degeneration of the ACL were excluded. Individuals were included in the study if they had had complete MRIs of the affected knee at baseline and at 4-year follow-up, had thigh MRIs at baseline and had no additional surgery in the meantime.

### Imaging: Plain radiographs

Bilateral standing posterior-anterior knee radiographs were acquired for all subjects in the OAI. Additional details on radiograph acquisition in OAI are available at http://oai.epi-ucsf.org/datarelease/OperationsManuals.asp. Kellgren-Lawrence (KL) scores at baseline were obtained from the OAI database.

### Imaging: Knee MRI

MR images were acquired at 4 clinical sites using 3T MRI scanners (Siemens Magnetom Trio; Siemens, Erlangen, Germany). Identical coils were used for all studies at all scanners. Bilateral knee MR images, obtained using quadrature transmit-receive knee coils (USA Instruments, Aurora, OH), included coronal 2D intermediate (IM)-weighted (w) fast spin-echo (FSE) sequences (TE 29ms, TR 3700ms, slice thickness 3mm, number of slices 35, field of view (FOV) 140mm, matrix 307x384, bandwidth 352 Hz/pixel), sagittal 2D IM-w FSE sequences with fat suppression (FS) (TE 30ms, TR 3200ms, slice thickness 3mm, number of slices 37, FOV 160mm, matrix 313x448, bandwidth 248Hz/pixel) and sagittal 3D dual-echo in steady state (DESS) sequences with selective water excitation (WE) with coronal and axial reformations (TE 4.7ms, TR 16.3ms, flip angle 25°, slice thickness 0.7mm, number of slices 160, FOV 140mm, matrix 307x384, bandwidth 185Hz/pixel). For MR imaging of the thigh, the patellar apex was palpated and the mid thigh region was defined as 150mm above the patellar apex. Axial T1-w scans (TE 13ms, TE 600ms, slice thickness 5mm, number of slices 15, FOV 500mm, matrix 384x512, bandwidth 199 Hz/pixel) were acquired with the bottom slice positioned at the medial femoral growth plate. Details of the acquisition protocols have been published (www.oai.ucsf.edu) [21].

### WORMS

Knee MR images were reviewed on picture archiving communication system (PACS) workstations (Agfa, Ridgefield Park, NJ). Knee MRIs were assessed morphologically for OA-related abnormalities by two musculoskeletal radiologists separately (P.M.J., L.N.; 7 and 9 years of experience) using the modified semi-quantitative whole-organ magnetic resonance imaging score (WORMS) [22, 23]; if scores were not identical consensus readings by both radiologists and a third experienced independent radiologist (T.M.L., 26 years of experience) were performed. A total WORMS score, with a maximum of 110 was calculated as the sum of grades for the individual knee structures: (i) meniscus abnormalities (score 0–4 for 6 locations; additionally scores of 0–6 were calculated for each meniscus [24]), (ii) cartilage lesions (score 0–6 in 6 regions), (iii) bone marrow lesions (score 0–3 in 6 regions) (iv) ligament abnormalities (score 0–4 in 6 locations), (v) effusion (0–3), (VI) intraarticular body (0–2) and (VII) baker cyst (0–3) [25].

### Thigh muscle cross-sectional area (CSA) measurements

**Fig 1:**
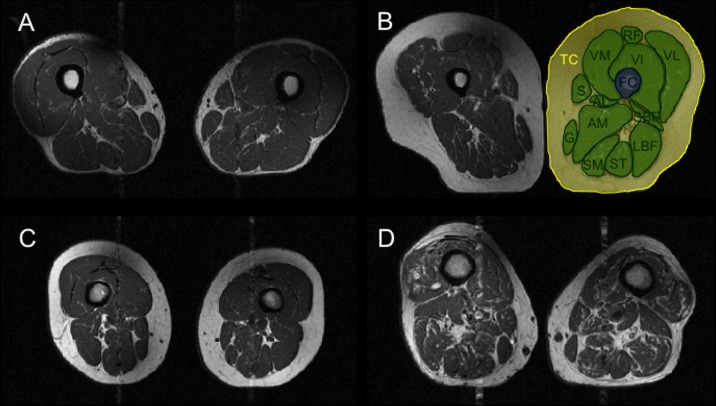
Segmentation on axial T1-w images of the thigh. Segmentation was performed for the vastus medius (VM), intermedius (IM) and lateralis (VL), rectus femoris (RF), sartorius (S), gracilis (G), adductor magnus (AM), adductor longus (AL), short head of biceps (SBF), long head of biceps (LBF), semitendinosus (ST), semimembranosus (SM), total thigh circumference (TC) and femur circumference (FC). Examples are given of four different individuals. (A) high muscle volume, high muscle/ fat ratio. (B) high muscle volume, small muscle/ fat ratio. (C) small muscle volume. (D) high fatty infiltration of thigh muscles.

In three central sections (images 7/15 to 9/15) of the thigh spline-based segmentation was performed by two radiologists (P.M.J., L.N.) with in-house developed software implemented in MATLAB (The Mathworks Inc., Natick, MA). To exclude body size as a confounding factor, allometric scaling of muscle cross-sectional area (CSA, m^2^) using body surface area (BSA, m^2^) was performed as described previously (corrected muscle CSA = uncorrected muscle CSA / BSA^2/3^) [14, 26]. BSA was calculated using the mosteller method (BSA (m^2^) = (height (cm) x weight (kg) /3600)^1/2^). The quadriceps (rectus femoris, vastus lateralis (VL), vastus medialis (VM), vastus intermedius), adductors (adductor longus, adductor magnus), hamstrings (semimembranosus, semitendinosus, short head and long head of biceps femoris), sartorius and gracilis muscles, the total thigh and the femoral bone were segmented (Fig 1). CSA was calculated for the parameters total muscle CSA, quadriceps CSA and hamstring CSA. VL/ VM ratio and muscle/ fat ratio were calculated additionally (extramuscular fat including vessles and nerves and other structures; CSA_extramuscular fat_ = CSAtotal thigh—CSA_bone_—CSA_total muscle_; muscle/ fat ratio = CSA_total muscle_ / CSA_extramuscular fat_; VL/ VM ratio = CSA_VL_ / CSA_VM_).

### Muscle Fat infiltration

Thigh MRIs were reviewed on PACS workstations (Agfa, Ridgefield Park, NJ). Fatty infiltration was graded using a five-point semi-quantitative scale (Goutallier score) described by Goutallier et al and modified by Fuchs et al [27–29]: Grade 0, normal; grade 1, some fatty streaks; grade 2, < 50% fatty infiltration of the muscle; grade 3, as much fat as muscle; grade 4, >50% fatty infiltration of the muscle. All MRI studies were interpreted in consensus by two radiologists (P.M.J., L.N.). A third radiologist (T.M.L.) was consulted in case of disagreement.

### WOMAC

Knee symptoms were assessed using WOMAC (University of Western Ontario and the McMaster University in Canada) scores [30, 31].

### PASE

Physical activity levels were measured using the Physical Activity Scale for the Elderly (PASE), a well-established and reliable questionnaire that has been validated in both older and younger individuals [32, 33].

### Isometric strength

Isometric knee flexion and extension strength measurements were performed using a *Good Strength apparatus (Metitur*, *Jyväskylä*, Finland; www.oai.ucsf.edu/datarelease/OperationsManuals.asp*)*. Two submaximal practice trials were completed. Then strength (N) was measured three times for 3 seconds, each separated by 30 seconds; the highest value for a limb is used for maximal strength reported.

### Statistical analysis

Statistical processing was performed with SPSS version 17.0 (SPSS Institute, Chicago, IL, USA) (P.M.J., T.B.). All tests were performed based on a 0.05 level of significance. Pearson correlations were used for correlation analyses of the different muscle characteristics. Partial Spearman correlations, adjusting for the covariates, were used for correlation analyses of different knee OA parameters with muscle characteristics. Multiple linear regression models, controlling for covariates, were used for comparisons between the different ACL groups. Additional multiple linear regression analyses were performed by including each muscle characteristic in the model. From the regression models we obtained adjusted means and standard errors (SEM) and 95% confidence intervals, if not stated otherwise. Covariates were OA risk factors including age (years), gender and body mass index (BMI; calculated as weight in kg/height m squared).

### Reproducibility

Interreader-reproducibility for CSA measurements was calculated for each muscle separately and for the total thigh muscle. The coefficient of variation (CV) was determined using root-mean-square averages of standard deviations of repeated measurements as described previously [34]. Good reproducibility in our research group for WORMS assessment was reported previously [14, 17, 35, 36].

## Results

Subject characteristics are given in Table 1 and in S1 Appendix.

**Table 1:** Subject characteristics.

Group/ Subgroup	Number of individuals (right/ left)	Age (years)	Gender (m/ f)	BMI (kg/ m^2^)	BSA (m^2^)	PASE	KL score
Total cohort	54	57.8±10.6	23 / 31	27.3±4.4	1.9±0.3	164±90	2.0±1.2
Normal ACL	24 (24/0)	64.0±9.7	6 / 18	26.7±4.2	1.8±0.2	144±85	1.8±1.0
ACL rupture	15 (7/8)	56.8±10.2	8 / 7	29.2±5.0	2.0±0.3	155±69	2.4±1.2
ACL reconstruction	15 (5/10)	48.7±2.4	9 / 6	26.5±3.9	1.9±0.2	204±107	1.9±1.3
Normal ACL vs ACL Rupture		0.032	0.076	0.098	0.049	0.693	0.079
Normal ACL vs ACL Reconstruction		<0.001	0.029	0.895	0.289	0.062	0.624
ACL Rupture vs ACL Reconstruction		0.006	0.724	0.110	0.396	0.147	0.309

Mean ± SD are given.

ACL, Anterior Cruciate Ligament; m, male; f, female; KL, Kellgren & Lawrence; BMI, body mass index; BSA; body surface area; PASE, physical activity scale of the elderly

### WORMS and ACL status

Subjects with ACL ruptures had worse total WORMS scores (mean±SEM, 44.1±3.5; 95% CI, 27.1, 51.0) than subjects with ACL reconstruction (30.8±4.0, 22.8, 38.8; P = 0.015) and subjects with normal ACL (21.3±3.0, 15.2, 27.4; P<0.001) (Table 2). The ACL rupture group had more severe scores for cartilage, meniscus and BME. Cartilage scores were worse in knees with ACL ruptures in all femorotibial compartments compared with subjects with knees normal ACL (all P<0.05). Knees with ACL reconstruction had worse cartilage scores at the medial femoral condyle as compared to subjects with normal ACL (MFC; 2.7±0.5 vs 1.0±0.3, P = 0.011). Knees with ACL reconstruction had worse progression at the medial tibia plateau (MT; 1.0±0.2 vs 0.3±0.2, P = 0.027). All other comparisons of WORMS progression scores did not show significant differences between the ACL groups.

**Table 2:** WORMS scorings.

**A**	**Normal ACL**	**ACL rupture**	**P** ^**a**^	**ACL reconstruction**	**P** ^**b**^	**P** ^**c**^
**BASELINE**						
Total score	21.3±3.0 (15.2, 27.4)	44.1±3.5 (37.1, 51.0)#*	<0.001*	**30.8±4.0 (22.8, 38.9)#**	0.096	**0.015#**
Cartilage	9.5±1.4 (6.7, 12.3)	17.6±1.6 (14.4, 20.8)*	0.001*	13.1±1.8 (9.4, 16.8)	0.173	0.071
MM	1.8±0.3 (1.1, 2.4)	3.7±0.4 (2.9, 4.5)*	0.001*	3.1±0.4 (2.2, 4.0)*	0.036*	0.336
LM	1.7±4.4 (0.8, 2.6)	2.8±0.5 (1.8, 3.8)	0.12	1.3±0.6 (0.1, 2.5)	0.627	0.059
BME	2.4±0.7 (1.1, 3.8)	6.2±0.8 (4.7, 7.8)#*	0.001*	**3.2±0.9 (1.4, 5.0)#**	0.545	**0.014#**
PAT	2.5±0.4 (1.8, 3.2)	2.8±0.4 (2.0, 3.6)	0.636	1.9±0.5 (1.0, 2.8)	0.333	0.15
Trochlea	2.5±0.3 (1.7, 3.0)	3.0±0.4 (2.2, 3.7)	0.216	2.1±0.4 (1.3, 3.0)	0.722	0.14
MFC	1.0±0.3 (0.3, 1.7)	3.4±0.4 (2.6, 4.2)*	<0.001*	2.7±0.5 (1.8, 3.6)*	0.011*	0.262
LFC	0.9±0.4 (0.2, 1.6)	2.3±0.4 (1.4, 3.1)	0.02	2.0±0.5 (1.0, 3.0)	0.114	0.655
MT	1.2±0.4 (0.5, 1.9)	3.1±0.4 (2.3, 3.9)*	0.001*	2.0±0.5 (1.1, 3.0)	0.198	0.091
LT	1.5±0.4 (0.7, 2.3)	3.0±0.4 (2.1, 3.9)*	0.014*	2.3±0.5 (1.2, 3.3)	0.276	0.267
**B**	**Normal ACL**	**ACL rupture**	**P** ^**a**^	**ACL reconstruction**	**P** ^**b**^	**P** ^**c**^
**PROGRESSION**						
Total score	6.3±1.2 (4.0, 8.7)	6.7±1.3 (4.1, 9.4)	0.809	7.6±1.5 (4.5, 10.7)	0.543	0.667
Cartilage	2.9±0.5 (1.9, 4.0)	3.4±0.6 (2.2, 4.6)	0.594	3.7±0.7 (2.3, 5.1)	0.454	0.749
MM	0.3±0.1 (0.1, 0.6)	0.4±0.2 (0.1, 0.7)	0.869	0.3±0.2 (-0.1, 0.7)	0.886	0.765
LM	0.1±0.1 (-0.2, 0.4)	0.4±0.2 (0.1, 0.7)	0.203	0.6±0.2 (0.2, 1.0)	0.081	0.463
BME	1.1±0.3 (0.2, 1.9)	0.8±0.5 (-0.2, 1.7)	0.662	0.8±0.5 (-0.3, 1.9)	0.697	0.982
PAT	0.7±0.2 (0.3, 1.0)	0.4±0.2 (-0.0, 0.8)	0.255	0.6±0.2 (0.2, 1.1)	0.934	0.351
Trochlea	0.3±0.2 (-0.0, 0.7)	0.6±0.2 (0.2, 1.0)	0.301	0.8±0.2 (0.4, 1.3)	0.093	0.382
MFC	0.7±0.2 (0.3, 1.2)	0.5±0.2 (0.1, 1.0)	0.524	0.5±0.3 (-0.0, 1.1)	0.597	0.985
LFC	0.5±0.2 (0.1, 0.9)	1.0±0.2 (0.6, 1.4)	0.092	0.5±0.2 (-0.0, 0.9)	0.89	0.098
MT	0.3±0.2 (-0.1, 0.6)	0.5±0.2 (0.1, 0.9)	0.319	1.0±0.2 (0.5, 1.5)*	0.027*	0.136
LT	0.4±0.1 (0.2, 0.7)	0.4±0.1 (0.1, 0.7)	0.769	0.2±0.2 (0.1, 0.6)	0.369	0.488

Mean±SEM (lower, upper 95% CI) are given for the different groups, controlling for the co-variates age, gender and BMI. When controlling for KL-scores additionally, P-values for the difference of MM scores between the “normal ACL” and the “ACL reconstruction” group and for the difference of the LT cartilage scores between the “normal ACL” group and the “ACL rupture” group lost significance (P>0.05). All other significant differences (P<0.05) remained significant after additional controlling for KL-scores.

(A) Baseline. (B) 4-year Progression (4-year change).

SEM, standard error of the mean; CI, confidence interval; WORMS, whole-organ MR imaging score; MM, medial meniscus; LM, lateral meniscus; BME, bone marrow edema like lesion; PAT, patellar cartilage; Trochlea, trochlear cartilage; MFC, medial femoral condyle cartilage; LFC lateral femoral condyle cartilage; MT, medial tibia plateau cartilage; LT, lateral tibia plateau cartilage; ACL, anterior cruciate ligament;

^a^ P-values for the difference between values for the “normal ACL” group and the “ACL rupture” group. Values are in **bold** writing and are marked with * if significantly different from the values in the “normal ACL” group.

^b^ P-values for the difference between values for the “normal ACL” group and the “ACL reconstruction” group. Values are in **bold** writing and are marked with * if significantly different from the values in the “normal ACL” group.

^c^ P-values for the difference between values for the “ACL reconstruction” group and the “ACL rupture” group. Values of the “ACL rupture” group and the “ACL reconstruction” group are in **bold** writing and marked with **#** if significantly different.

### WORMS and muscle characteristics

**Fig 2:**
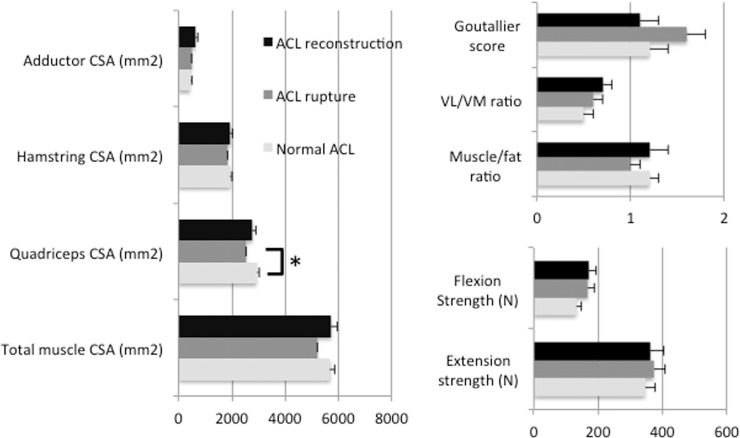
Muscle characteristics. Mean±SEM are given for the different ACL groups, controlling for the co-variates age, gender and BMI. ACL, anterior cruciate ligament; CSA, cross-sectional area; VL, vastus lateralis muscle; VM, vastus medialis muscle. *P<0.05.

Associations between muscle characteristics (Pearson correlations) and differences of muscle characteristics between the ACL groups are given in Table 3 and Fig 2. For the entire study cohort, higher muscle CSA correlated significantly with lower WORMS total scores (Partial Spearman correlation, rho = -0.38, P = 0.007; Table 4) and with lower cartilage scores (rho = -0.40, P = 0.003), indicating less degenerative changes. More severe cartilage defects at the lateral tibia plateau (LT; rho = -0.35, P = 0.12), lateral femoral condyle (LFC; rho = -0.38, P = 0.006) and trochlea (rho = -0.35; P = 0.013) correlated significantly with smaller total muscle CSA. P-values were the smallest for the quadriceps CSA. High muscle/ fat ratio was associated with low WORMS scores (rho = -0.31; P = 0.026; cartilage score, rho = -0.35, rho = 0.013). High Goutallier scores correlated significantly with high total WORMS scores (rho = 0.38, P = 0.005), high lateral meniscus (LM) scores (rho = 0.33, P = 0.018) and high cartilage scores (rho = 0.35, P = 0.012), in particular at the LT (rho = 0.43, P = 0.002), LFC (rho = 0.38, P = 0.005) and trochlea (rho = 0.41, P = 0.003). Flexion and extension strength did not show any significant correlation with WORMS scores (P>0.05). There was no significant correlation between muscle characteristics and WORMS progression over 4 years (data not shown).

**Table 3:** Pearson correlations.

Parameter	Total muscle CSA	VL/VM ratio	Muscle/fat ratio	Goutallier score	Extension strength	Flexion strength	BMI
Total muscle CSA	-						
VL/VM ratio	R = 0.17	-					
Muscle/fat ratio	**R = 0.72**	R-0.05	-				
Goutallier score	R = -0.06	R = -0.00	**R = -0.30**	-			
Extension strength	**R = 0.45**	R = -0.08	**R = 0.46**	R = -0.02	-		
Flexion strength	**R = 0.51**	R = 0.00	**R = 0.50**	R = 0.03	**R = 0.86**	-	
BMI	**R = 0.50**	R = -0.19	R = 0.05	**R = 0.32**	R = 0.15	R = 0.16	-
PASE	R = 0.16	R = -0.04	R = 0.26	**R = -0.33**	**R = 0.30**	**R = 0.32**	**R = -0.27**

R-values for the correlations between muscle characteristics, PASE and BMI. If P-values were between 0.01 and 0.05, R-values are in **bold** text, if P-values were <0.01, R-values are in bold text and **underlined.**

PASE, Physical Activity Scores of the Elderly; BMI, Body Mass Index; VL, vastus lateralis muscle; VM, vastus medialis muscle.

**Table 4:** Partial spearman correlations of muscle characteristics.

**A**	**Total score**	**Cartilage**	**MM**	**LM**	**BME**	
**BASELINE**						
total thigh muscle CSA (mm2)	**rho = -0.38**	**rho = -0.40**	rho = -0.16	rho = -0.25	rho = -0.24	
Quadriceps CSA (mm2)	**rho = -0.40**	**rho = -0.43**	rho = -0.11	rho = -0.28	rho = -0.25	
Hamstring CSA (mm2)	rho = -0.06	rho = -0.03	rho = -0.14	rho = -0.05	rho = -0.04	
Adductor CSA (mm2)	rho = -0.25	**rho = -0.31**	rho = -0.07	rho = -0.16	rho = -0.16	
Muscle/fat ratio	**rho = -0.31**	**rho = -0.35**	rho = -0.15	rho = -0.17	rho = -0.21	
VL/VM ratio	rho = -0.16	rrho = -0.21	rho = 0.05	rho = -0.18	rho = -0.05	
Goutallier score	**rho = 0.38**	**rho = 0.35**	rho = 0.11	**rho = 0.33**	rho = 0.23	
Extension strength (N)	rho = -0.07	rho = -0.15	rho = 0.22	rho = -0.12	rho = -0.10	
Flexion Strength (N)	rho = -0.08	rho = -0.18	rho = 0.09	rho = -0.06	rho = -0.08	
**B**	**PAT**	**Trochlea**	**MFC**	**LFC**	**MT**	**LT**
**BASELINE**						
total thigh muscle CSA (mm2)	rho = -0.17	**rho = -0.35**	rho = -0.21	**rho = -0.38**	rho = -0.22	**rho = -0.35**
Quadriceps CSA (mm2)	rho = -0.18	**rho = -0.31**	rho = -0.23	**rho = -0.43**	rho = -0.26	**rho = -0.36**
Hamstring CSA (mm2)	rho = 0.07	rho = -0.07	rho = -0.05	rho = -0.05	rho = -0.03	rho = 0.00
Adductor CSA (mm2)	rho = -0.19	**rho = -0.32**	rho = -0.12	rho = -0.25	rho = -0.11	**rho = -0.33**
Muscle/fat ratio	rho = -0.21	**rho = -0.29**	rho-0.26	rho = -0.24	rho = -0.24	rho = -0.22
VL/VM ratio	rho = -0.15	rho = -0.18	rho = 0.06	rho = -0.24	rho = -0.17	rho = -0.22
Goutallier score	rho = 0.15	**rho = 0.41**	rho = 0.09	**rho = 0.38**	rho = 0.03	**rho = 0.43**
Extension strength (N)	rho = -0.11	rho = -0.10	rho = -0.05	rho = -0.17	rho = 0.00	rho = -0.20
Flexion Strength (N)	rho = -0.15	rho = -0.14	rho = -0.12	rho = -0.10	rho = -0.18	rho = -0.08

(A) WORMS scores (controlling for the co-variates age, gender and BMI) and (B) WORMS cartilage scores in different knee compartments (controlling for the co-variates age, gender and BMI). If P-values were between 0.01 and 0.05, rho-values are in **bold** text, if P-values were <0.01, rho-values are in bold text and **underlined.**

WORMS, whole-organ MR imaging score; MM, medial meniscus; LM, lateral meniscus; BME, bone marrow edema like lesion; PAT, patellar cartilage; Trochlea, trochlear cartilage; MFC, medial femoral condyle cartilage; LFC lateral femoral condyle cartilage; MT, medial tibia plateau cartilage; LT, lateral tibia plateau cartilage; ACL, anterior cruciate ligament; CSA, cross-sectional area; VL, vastus lateralis muscle; VM, vastus medialis muscle.

### Multiple regression models

Including each muscle characteristic in the multiple regression model revealed similar results as compared to the separate analyses. In the baseline analyses, ACL ruptures were associated with worse WORMS scores (P<0.001). ACL reconstruction was associated with increased cartilage degeneration at the MFC (P = 0.011). In the progression analysis, ACL reconstruction was associated with an increased progression of cartilage degeneration at the MT (P = 0.029). Also for the influence of muscle characteristics, the results from the multiple regression models were similar to the separate analyses. An additional significant correlation was found for higher “flexion strength” with less cartilage degeneration at baseline (P = 0.041; medial tibia, P = 0.049) and for more severe Goutallier scores with an increased progression of cartilage degeneration at the patella (P = 0.047) in the longitudinal analysis.

### WOMAC

The mean WOMAC total score of the entire study cohort was 17.1±2.6 (mean±SEM). The differences for WOMAC scores between the ACL groups were not significant (all P>0.05; normal ACL, 20.9±4.4; ACL rupture 14.8±5.0; ACL reconstruction 13.4±5.8). Extension strength (rho = -0.35, P = 0.015) and flexion strength (rho = -0.389, P = 0.006) correlated significantly with WOMAC scores. All other muscle parameters had no significant correlations with WOMAC scores (P>0.05; data not shown).

### Time since injury

The mean follow-up time after ACL reconstruction was 11.4±8.2 years (mean ± standard deviation (SD)). The mean time after the first injury of the analyzed knee was 21.9±12.9 years for the “ACL rupture” group (information available for 13/15 individuals) and 18.0±10.9 years for the “ACL reconstruction” group (information available for 14/15 individuals). This difference was not significant (P = 0.409). The “time after first injury” was significantly positively correlated with total WORMS scores (rho = 0.45, P = 0.028), MM scores (rho = 0.48, P = 0.017) and cartilage scores (rho = 0.42, P = 0.039). There were no significant correlations with muscle parameters or WOMAC scores (P>0.05).

When comparing WORMS scores of the “ACL rupture” and the “ACL reconstruction” group and adjusting for the parameter “time since first injury” in a multiple regression model, the differences in total WORMS scores and BME scores remained significant (P = 0.013 and P = 0.019, respectively). When adjusting for all co-factors (age, gender, BMI) plus for “time since first injury” in a multiple regression model, significant differences were eliminated (P>0.05).

### Reproducibility

The CV for total thigh muscle CSA measurements was 0.7% (171 mm^2^) and varied between 0.2% and 4.0% (6 mm^2^ and 46 mm^2^) for the individual muscle CSA measurements.

## Discussion

In this study morphological knee abnormalities and muscle characteristics in individuals with ruptured, reconstructed and normal ACL were evaluated in a baseline and a 4 year progression analysis of the OAI using 3T MRI. Worse baseline WORMS scores were found in subjects with ACL ruptures. Worse cartilage scores at the MFC and a statistical trend for increased WORMS total scores were found in subjects with ACL reconstruction, independent of muscle characteristics. In the progression analysis, ACL reconstruction was associated with an increased progression of cartilage degeneration at the MT. These results suggest, that (i) subjects with chronic ACL tears suffer from more advanced OA than subjects with ACL reconstruction, (ii) medial OA progresses despite ACL reconstruction and (iii) that high thigh muscle CSA, high muscle/ fat ratio and low muscle fat infiltration have a possible protective effect against the prevalence of knee OA.

Long-term studies report good results ten or more years after ACL surgery [6]. However, there is no definite evidence, that surgical treatment is superior to non-surgical treatment regarding the prevention of OA [37–39]. Cartilage degeneration and other degenerative changes at the knee progress despite functionally stable ACL reconstruction [8, 9, 15, 40, 41]. Degenerative cartilage changes appear seven years after surgery and about 10–17 years after the injury about 50% of patients develop OA [6, 35, 42–50]. There are only few studies that compare surgical versus non-surgical treatment of ACL injuries in an over 10 year follow-up and most studies only performed conventional radiographs for radiological outcome evaluation [43, 45, 51]. Meunier et al found similar subjective outcome and similar rates of radiographic OA, but increased clinical instability in non-operatively treated subjects 15 years after injury [52]. In a 20 year follow-up, Louboutin et al found a lower percentage of radiographic OA in surgically treated patients [15], which is in line with our findings.

In our MR study, knees with ACL rupture showed significantly more degenerative changes. In case of ACL reconstruction, degeneration was found primarily in the medial femorotibial compartment for meniscus and cartilage scores, but not in the lateral compartment. This observation is in line with previous studies, that reported an increased degeneration in the medial femorotibial compartment after ACL reconstruction as opposed to the initial traumatic injury of the lateral femorotibial compartment [19, 53–57]. This may be due to not entirely restored kinematics, a persisting rotational instability and persisting anterior-posterior tibial translation after ACL reconstruction [2, 55, 58].

Previous studies reported, that thigh muscle volume and VL/ VM ratio are associated with knee OA [14]. We found that high thigh muscle CSA is associated with less knee OA changes in subjects with ACL injuries. The quadriceps muscle plays a particularly important role with respect to knee OA [10, 11]. In contrast to the findings of Pan et al [14], VL/ VM ratio had no major influence on OA in our study cohort. Reasons may be, that we did not categorize the parameter, that most of our subjects were from the OAI progression cohort and that our study cohort was smaller. With respect to strength measurements, flexion strength showed a significant influence on cartilage scores in the multivariate regression model. In addition, strength and clinical WOMAC scores correlated significantly. Also other studies reported, that muscle weakness may be a risk factor for OA and that improving muscle strength improves the outcome after ACL reconstruction [10, 11, 59]. In our study, the influence of muscle characteristics on OA progression was not significant; only minor differences were depicted (Tables 2 and 4). This may be due to a small study cohort, a too short longitudinal follow-up or ceiling effects of WORMS parameters.

One major limitation of this study is the missing information on timing of the ACL rupture in relation to OAI baseline. However, most patients recalled a severe injury of the assessed knee joint and the difference in time between the first injury and the baseline of the study was not significantly different between the “ACL rupture” and the “ACL reconstruction” group. The importance of the parameter “time since first injury” was demonstrated, since it was associated with more severe knee joint degeneration. Although most patients recalled a severe knee injury, the exact origin of ACL ruptures in both, the “ACL rupture” and the “ACL reconstruction” group is not known. This missing information was one reason for performing a longitudinal progression analysis. The impact of ACL reconstruction versus chronic ACL rupture on long-term longitudinal progression of OA has not yet been evaluated [19]. However, most significant differences were found for baseline values. Particularly in subjects with prevalent OA, progression analysis is challenging [60]. Further, we were not able to differentiate between different surgical techniques for ACL reconstruction. It remains unclear whether muscle parameters had an influence on morphological knee OA or vice versa. Additionally, the groups we analyzed turned out to be relatively small and heterogeneous regarding age, gender and BMI after application of the inclusion and exclusion criteria, mainly availability of MR scans at baseline and at 4-year follow-up. Therefore we included these parameters as co-variates in the analyses. Power-analyses revealed that a total sample size of n = 30 was required for total WORMS scores and a total sample size of n = 54 was required for assessment of total muscle CSA. Still, the screening process and the analysis of knee and thigh MR images in this detailed fashion is extremely time-consuming and assessment of larger cohorts is aim of the ongoing osteoarthritis initiative.

In summary, in this 3T MRI study, ACL ruptures were associated with an increased prevalence of degenerative changes at the knee as compared to ACL reconstruction and as compared to knees with normal ACL. Knees with ACL reconstruction had more degenerative changes in the medial femorotibial compartment as compared to knees with normal ACL and showed an increased progression of cartilage degeneration at the MT. The results support the assumption of a lower risk for OA in knees with ACL reconstruction as compared to knees with chronic ACL ruptures, but patients still suffer from an increased risk for early OA, particularly of the medial femorotibial compartment, as compared to knees with normal ACL. Muscle parameters had an additional influence on the prevalence of OA. High muscle CSA had a moderate but significant association with reduced OA changes, independent of the ACL status. However, further progression of advanced OA may not be halted. In conclusion, the results might be helpful for patient education regarding the potential influence of physiotherapy and live-style interventions aiming BMI and body fat content reduction on their possible course of disease.

## Supporting Information

S1 Appendix (PDF)
