# Supplementary material for: Degeneration in ACL Injured Knees with and without Reconstruction in Relation to Muscle Size and Fat Content—Data from the Osteoarthritis Initiative
Source: PLoS One. 2016 Dec 5;11(12):e0166865. doi: 10.1371/journal.pone.0166865 (PMC5137877; doi:10.1371/journal.pone.0166865)
Supplement: S1 Appendix — (PDF) [file pone.0166865.s001.pdf]

ACL_3repair_	SideR1_L2	YearsAfterRec	Yearsafterfirst	Goutallier	MMscore0Y	LMscore0Y
3	1	6	21	2	2	5
3	1	12	10	2	2	1
3	2	4	2	1	0	1
3	2	4	10	1	3	0
3	2	7	5	2	4	0
3	2	9	26	2	4	1
3	2	9	7	1	4	0
3	2	10	37	1	1	0
3	2	12	10	0	2	4
3	2	21	22	1	5	0
3	2	17	24	1	6	0
3	2	17	18	0	4	0
3	1	2	28	2	6	1
3	1	34	33	1	5	0
3	1	10		1	1	0
2	1		10	2	5	6
2	1		19	2	3	3
2	2		20	2	3	0
2	2		24	2	4	5
2	2		33	1	6	2
2	2		29	2	2	4
2	2		30	2	5	1
2	2		38	3	4	6
2	2			2	5	1
2	1		5	1	1	1
2	1			1	6	1
2	1		28	0	3	0
2	1		2	1	2	6
2	1		41	2	4	3
2	2		6	2	1	2
1	1			1	1	0
1	1			1	1	0
1	1			1	2	3
1	1			0	4	3
1	1			1	0	0
1	1			2	1	0
1	1			1	4	1
1	1			3	2	3
1	1			1	1	1
1	1			2	1	0
1	1			2	1	0
1	1			2	1	3
1	1			1	1	1
1	1			1	0	0
1	1			2	1	6
1	1			1	0	2
1	1			0	3	0
1	1			1	4	3
1	1			1	4	4
1	1			3	3	6
1	1			2	1	1
1	1			2	1	4
1	1			1	1	4
1	1			2	3	3

SUMlig0Y	CartSUM0Y	BMESUM0Y	Jointeffusion0	Intraartbody0	bakercyst0Y	SUM0Yothers
3	15	6	1	0	0	1
8	10	0	1	0	0	1
2	5	2	0	0	0	0
3	8	0	1	0	1	2
2	13	2	1	0	1	2
5	5	0	1	0	0	1
7	14	5	0	0	0	0
4	3	0	0	0	0	0
6	19	6	1	0	2	3
9	12	3	1	0	0	1
2	11	3	0	0	1	1
2	6	1	0	0	0	0
6	22	4	2	0	2	4
3	19	4	1	1	0	2
1	16	2	0	0	2	2
9	18	2	1	0	3	4
5	9	1	0	0	0	0
5	14	5	0	0	1	1
4	25	10	1	0	0	1
8	18	7	1	0	0	1
8	21	7	0	0	1	1
7	11	2	1	0	0	1
8	25	12	2	0	2	4
8	18	5	1	0	0	1
9	13	9	2	0	0	2
9	23	12	2	0	2	4
4	13	2	1	0	0	1
7	19	8	1	0	2	3
4	21	6	1	0	2	3
9	14	2	0	0	0	0
2	15	6	0	0	0	0
0	7	4	0	0	1	1
2	17	3	0	0	3	3
2	3	0	1	0	0	1
2	5	3	0	0	0	0
1	11	3	1	0	2	3
4	10	2	1	0	0	1
5	17	2	1	0	0	1
2	15	6	1	1	2	4
3	6	2	0	0	0	0
6	0	0	0	0	1	1
2	7	2	1	0	1	2
1	4	0	0	0	1	1
2	0	1	0	0	1	1
3	17	8	2	2	3	7
2	4	0	1	0	0	1
3	4	0	0	0	1	1
5	6	3	1	0	0	1
2	18	6	1	0	0	1
9	25	10	1	1	2	4
1	13	1	0	0	2	2
1	16	2	0	0	2	2
4	18	7	1	0	1	2
6	12	1	1	0	0	1

SUMSUMvalu	MMscoreIncre	LMscoreIncre	SUMMenScor	SUMligIncreas	CartSUMIncre	BMESUMIncre
37	0	1	1	4	4	0
24	0	0	1	-1	3	0
12	1	0	3	3	9	-2
18	0	0	0	0	1	0
26	-1	2	2	0	5	2
22	0	0	0	0	5	0
34	0	0	0	1	2	2
9	0	0	0	1	2	1
43	2	0	6	4	7	5
35	1	1	2	-2	0	3
26	0	0	0	1	4	4
14	0	0	0	0	1	0
50	0	2	3	1	3	3
38	1	1	2	4	0	-1
22	0	0	2	0	2	1
50	0	0	2	3	9	4
26	0	0	1	0	3	2
29	0	1	3	0	4	2
60	0	1	0	0	2	3
49	0	0	1	0	2	-2
45	1	0	3	0	2	2
31	1	0	1	1	2	2
67	0	0	0	0	3	-3
43	1	0	2	0	5	0
37	0	1	1	0	5	-2
60	0	2	5	-1	4	-2
26	1	0	1	1	3	4
51	0	0	0	2	1	-1
46	0	0	2	0	3	0
28	1	-1	1	2	0	2
24	1	0	1	0	4	1
13	0	0	0	1	0	0
35	1	0	1	2	4	3
17	0	0	1	1	5	0
12	1	1	0	0	5	0
20	0	0	0	1	2	3
25	0	0	1	0	2	0
33	0	0	2	3	3	0
30	0	-1	-1	1	2	1
13	0	0	0	0	2	2
9	0	0	0	-1	0	0
20	1	1	5	0	7	0
10	1	0	1	0	7	0
4	1	0	1	2	0	0
47	0	0	1	1	2	0
9	0	1	4	2	4	2
12	0	0	0	0	0	0
25	0	1	3	0	8	0
39	1	0	6	1	6	2
64	0	0	0	1	3	2
21	2	2	7	1	9	3
29	0	0	2	0	3	1
39	0	1	2	2	4	-1
29	0	0	0	2	3	1

JointeffusionIr	IntraartbodyIn	bakercystIncre	SUMothersInc	SUMSUMvalu	Gender	Age
0	0	0	0	9	1	46
0	0	0	0	3	1	50
0	0	0	0	13	2	49
0	0	0	0	1	1	48
0	0	0	0	9	1	49
0	0	0	0	5	1	49
1	0	0	1	6	2	50
0	0	0	0	4	1	50
0	1	1	2	24	1	55
0	0	0	0	3	2	49
0	0	0	0	9	1	47
1	0	0	1	2	1	49
-1	0	-1	-2	8	2	49
1	0	0	1	6	2	45
0	0	0	0	5	2	45
1	0	0	1	19	2	72
0	0	0	0	6	1	45
0	0	0	0	9	1	62
1	0	0	1	6	2	61
0	0	0	0	1	1	48
0	0	0	0	7	1	45
0	0	0	0	6	1	49
0	0	0	0	-1	1	58
0	0	0	0	7	1	52
-1	0	0	-1	3	2	70
0	0	0	0	6	2	69
0	0	0	0	9	2	45
0	0	1	1	3	2	66
0	0	0	0	5	1	65
1	0	1	2	7	2	45
1	0	1	2	8	2	66
0	0	0	0	1	2	46
1	0	-1	0	10	2	70
-1	0	0	-1	6	1	63
1	0	0	1	6	2	65
1	0	0	1	7	2	46
0	0	0	0	3	1	74
0	0	0	0	8	2	71
0	0	0	0	3	2	58
1	0	0	1	5	2	45
0	0	0	0	-1	2	77
1	0	2	3	15	2	70
0	0	0	0	8	2	69
0	0	0	0	3	2	75
0	0	0	0	4	2	72
0	0	0	0	12	2	46
0	0	0	0	0	1	58
0	0	0	0	11	1	74
0	0	0	0	15	2	61
0	0	0	0	6	1	65
0	0	0	0	20	2	69
1	0	0	1	7	2	67
0	0	1	1	8	2	66
-1	0	0	-1	5	1	65

BMI	PASE	FlexionMAXF	ExtensionMA	WOMACTotal	KL	QuadricepsBS
32	72	235	336	0	2	3,463.96
29	149	142	200	35	0	2,991.56
24	127	179	304	26	1	2,304.27
35	133	345	775	11	2	4,037.58
26	374	149	300	54	2	3,095.84
29	254	429	817	9	2	3,678.89
24	207	232	441	12	2	3,260.42
26	230	97	309	4	0	3,897.48
23	145	157	425	0	3	2,804.37
21	340	132	297	1	0	2,215.83
24	187	233	366	13	2	3,732.74
26	313	355	641	1	2	4,243.91
22	130	90	362	13	4	1,389.91
25	371	111	380	4	4	2,573.35
32	30	31	45	35	3	2,956.42
29	95	139	352	11	3	2,438.98
33	254	279	427	24	2	4,258.77
33	175	267	511	2	0	2,377.58
24	121	98	210	4	3	2,012.94
28	200	165	385	23	4	3,292.19
41	27	121	344	76	2	4,014.46
26	17	170	470	3	2	3,281.06
28	133	298	590	1	4	2,372.46
30	180			37	3	2,925.75
24	144	134	308	11	2	2,180.70
25	204	103	258	36	3	2,366.32
25	250	207	454	7	2	2,771.06
24	196	104	263	5	3	1,278.73
34	173	153	447	3	3	2,985.03
33	152	189	326	12	0	2,315.27
36	111	53	308	58	2	3,184.04
25	254			2	2	2,980.75
25	144	109	347	0	2	1,776.95
26	227	222	505	0	2	3,543.95
24	121	147	261	2	0	2,346.14
24	205	184	520	5	2	3,419.99
27	25	109	376	56	2	3,040.70
35	69	98	252	2	2	2,572.91
33	133	175	356	8	2	2,649.93
27	94	86	316	52	1	2,883.09
25	25	87	302	0	1	1,991.72
23	134	225	511	39	3	2,544.22
26	193	147	356	6	0	2,457.92
31	25	69	178	4	1	2,044.32
26	86	64	240	23	3	2,192.56
22	111	79	210	26	0	2,423.78
29	210	103	298	0	2	2,924.38
29	73	73	355	20	1	3,238.00
21	227	96	367	10	3	1,969.08
29	101	94	218	39	3	2,895.72
21	119	90	169	15	3	1,821.30
21	158	64	179	17	2	1,483.62
25	230	67	162	55	3	2,688.23
30	388	166	474	10	0	3,150.38

HamstringsBS	AdductorsBSA	VLVMratioBSA	TotalMuscleBS	TotalfatBSAad	MusclefatratioBSAadjusted
2,377.75	747.41	0.57	7,085.84	4,988.65	1.42
2,551.13	194.25	0.24	6,384.25	4,121.09	1.55
1,721.83	1,686.13	1.1	6,047.90	9,204.66	0.66
2,602.19	876.91	0.33	8,165.51	4,573.52	1.79
1,750.36	592.18	0.69	6,061.92	4,611.11	1.31
2,721.18	586.91	0.43	7,460.00	4,857.26	1.54
1,786.21	726.56	1.39	6,069.98	4,635.71	1.31
2,502.13	1,116.24	0.86	8,057.69	2,974.88	2.71
2,270.59	200.09	0.44	5,734.97	3,237.57	1.77
1,622.23	299.11	0.66	4,429.80	4,570.11	0.97
2,058.67	520.75	1.04	6,754.51	3,500.17	1.93
2,464.80	306.57	0.56	7,503.69	2,736.97	2.74
1,362.72	33.01	0.17	2,958.03	5,196.51	0.57
2,223.42	472.92	0.63	5,581.03	7,873.74	0.71
1,742.32	1,184.73	0.7	6,306.29	8,479.14	0.74
1,453.09	425.13	0.79	4,553.31	9,400.92	0.48
2,733.97	923.7	0.7	8,478.94	4,540.94	1.87
2,016.90	272.96	0.36	5,022.92	8,472.71	0.59
1,640.25	496.32	0.98	4,464.59	5,711.96	0.78
1,817.50	1,295.73	0.58	6,956.15	5,571.46	1.25
2,688.95	1,000.52	0.54	8,273.12	6,153.36	1.34
1,997.37	406.82	0.7	6,065.57	4,540.33	1.34
2,044.94	8.05	0.23	4,823.38	2,918.04	1.65
2,332.38	341.6	0.32	6,028.24	4,599.27	1.31
1,544.77	327.59	0.69	4,293.83	6,433.86	0.67
1,815.31	691.88	0.76	5,150.08	5,648.65	0.91
1,736.81	163.8	0.29	5,020.36	3,213.08	1.56
1,095.06	114.43	0.27	2,666.95	5,267.81	0.51
2,432.96	653.46	0.51	6,487.21	6,216.66	1.04
1,966.27	838.02	0.94	5,570.66	11,431.36	0.49
1,966.51	81.23	0.56	5,756.90	9,650.77	0.6
1,983.44	665.83	0.56	6,047.02	7,414.97	0.82
1,351.90	119.09	0.41	3,486.46	7,475.36	0.47
2,266.25	509.29	0.51	6,676.99	2,963.04	2.25
1,554.47	172.13	0.68	4,338.92	5,228.96	0.83
1,993.18	791.88	1.02	6,575.99	5,688.90	1.16
1,966.69	587.89	0.68	6,101.03	4,094.67	1.49
1,911.77	408.5	0.44	5,208.89	7,832.00	0.67
1,915.49	207.08	0.35	5,083.72	7,043.55	0.72
1,491.87	418.81	0.77	5,152.66	8,297.62	0.62
1,497.62	725.76	0.86	4,432.66	5,321.92	0.83
1,517.47	634.23	0.73	5,101.78	5,095.32	1
1,644.71	653.08	0.91	5,062.23	6,001.99	0.84
1,139.06	638.88	0.69	4,089.84	8,393.01	0.49
1,551.56	195.62	0.37	4,191.99	4,844.97	0.87
1,571.13	96.6	0.44	4,366.65	5,127.91	0.85
2,458.05	248.39	0.25	5,987.85	3,374.11	1.77
1,703.52	924	0.54	6,211.15	3,808.35	1.63
1,442.06	318.01	0.75	3,912.91	6,018.24	0.65
2,344.04	199.53	0.54	5,973.17	4,524.13	1.32
1,604.65	124.64	0.35	3,794.78	4,761.37	0.8
1,527.66	111.17	0.41	3,334.26	6,592.71	0.51
1,971.93	84.57	0.4	5,158.10	5,420.27	0.95
2,169.65	357.44	0.3	6,126.51	5,307.44	1.15
